# Supplementary material for: Comparative Proteome Profiling of Extracellular Vesicles from Three Growth Phases of Haematococcus pluvialis under High Light and Sodium Acetate Stresses
Source: Int J Mol Sci. 2024 May 16;25(10):5421. doi: 10.3390/ijms25105421 (PMC11121785; doi:10.3390/ijms25105421)
Supplement: Supplementary file 1 [file ijms-25-05421-s001.zip › Table S2 KEGG pathway analysis of significantly enriched DAPs from different comparison groups.pdf]

Supplemental Information

Proteome characterization of extracellular vesicles isolated from *Haematococcus pluvialis* with three stages under high light and high sodium acetate stresses

Qunju Hu[1], Yuanyuan Wang[1], Chaogang Wang[2*], Xiaojun Yan[1*]

Table S2 | KEGG pathway analysis of significantly enriched DAPs from different comparison groups.

Groups	HpEVs2 vs HpEVs1			HpEVs3 vs HpEVs2			HpEVs3 vs HpEVs1		
Regulation mode	Pathways	Number of DAPs	P-value	Pathways	Number of DAPs	P-value	Pathways	Number of DAPs	P-value
Combined	Photosynthesis - antenna proteins	7	0.000001	Citrate cycle (TCA cycle)	9	0.005790	Biosynthesis of unsaturated fatty acids	5	0.010127
	Oxidative phosphorylation	11	0.000063	Plant-pathogen interaction	6	0.028476	Ribosome	25	0.012970
	Photosynthesis	8	0.000376	Protein processing in endoplasmic reticulum	13	0.036913	Citrate cycle (TCA cycle)	10	0.017424
	Ribosome	13	0.001702				Fatty acid elongation	3	0.019960
	Monoterpenoid biosynthesis	2	0.001978				Protein processing in endoplasmic reticulum	17	0.030744
							Plant-pathogen interaction	7	0.042562
							Spliceosome	16	0.042833
Up-regulated	Ribosome	13	0.000001	Citrate cycle (TCA cycle)	8	0.000761	Citrate cycle (TCA cycle)	10	0.000412
	Monoterpenoid biosynthesis	2	0.000406	Protein processing in endoplasmic reticulum	11	0.004744	Spliceosome	15	0.000901
	Plant-pathogen interaction	3	0.0132077	Spliceosome	9	0.028741	Ribosome	18	0.006617
				Fatty acid elongation	2	0.029880	Glyoxylate and dicarboxylate metabolism	9	0.009190
				Glycine, serine and	5	0.030533	Protein processing in	13	0.010373

				threonine metabolism			endoplasmic reticulum		
				Various types of N-glycan biosynthesis	3	0.035682	Lysine degradation	2	0.019189
							Glycine, serine and threonine metabolism	6	0.037108
							Pyruvate metabolism	9	0.044069
							Valine, leucine and isoleucine degradation	4	0.045283
Down-regulated	Photosynthesis - antenna proteins	7	0.000000	Aminoacyl-tRNA biosynthesis	6	0.016448	Photosynthesis	9	0.000230
	Oxidative phosphorylation	11	0.000000	Phosphatidylinositol signaling system	3	0.026032	Oxidative phosphorylation	10	0.001305
	Photosynthesis	8	0.000004	Circadian rhythm - plant	1	0.039028	alpha-Linolenic acid metabolism	4	0.001834
	Taurine and hypotaurine metabolism	1	0.048049	Basal transcription factors	1	0.039028	Photosynthesis - antenna proteins	4	0.003662
				Other types of O-glycan biosynthesis	3	0.040171	Biosynthesis of unsaturated fatty acids	3	0.017484
							Fatty acid degradation	4	0.018662
							beta-Alanine metabolism	3	0.028062
